# Pre-emptive spectral graph protection strategies on multiplex social networks

**DOI:** 10.1007/s41109-018-0061-8

**Published:** 2018-04-11

**Authors:** Arie Wahyu Wijayanto, Tsuyoshi Murata

**Affiliations:** Department of Computer Science, School of Computing, Tokyo Institute of Technology, Tokyo, Japan

**Keywords:** Multiplex networks, Graph mining, Epidemic contagion, Node immunization

## Abstract

Constructing effective and scalable protection strategies over epidemic propagation is a challenging issue. It has been attracting interests in both theoretical and empirical studies. However, most of the recent developments are limited to the simplified single-layered networks. Multiplex social networks are social networks with multiple

layers where the same set of nodes appear in different layers. Consequently, a single attack can trigger simultaneous propagation in all corresponding layers. Therefore, suppressing propagation in multiplex topologies is more challenging given the fact that each layer also has a different structure. In this paper, we address the problem of suppressing the epidemic propagation in multiplex social networks by allocating protection resources throughout different layers. Given a multiplex graph, such as a social network, and *k* budget of protection resources, we aim to protect a set of nodes such that the percentage of survived nodes at the end of epidemics is maximized. We propose MultiplexShield, which employs the role of graph spectral properties, degree centrality and layer-wise stochastic propagation rate to pre-emptively select *k* nodes for protection. We also comprehensively evaluate our proposal in two different approaches: multiplex-based and layer-based node protection schemes. Furthermore, two kinds of common attacks are also evaluated: random and targeted attack. Experimental results show the effectiveness of our proposal on real-world datasets.

## Introduction

Real-world networks reveal the existence of multiple levels relationships. For instance, in social networks, an individual can possess membership of several communities which range in different functionalities from intimate (e.g., families, friends, clubs) to more serious (e.g., businesses, schools). In social networks, one can categorize edges based on the nature of the relationships (i.e., ties) or actions that they represent (Kivela et al. 2014). Reducing a social system to a network in which actors are connected in a pairwise fashion by only a single type of relationship is often a crude approximation of reality. Furthermore, the current insights in complex network analysis does not only consider networks as isolated graphs, but also characterizes how a network interacts with other networks and how this interaction affects epidemic spreading that occur on top of them (Kivela et al. 2014).

Multiplex social networks are social networks with multiple layers where the same set of nodes appears in different layers (Abraham et al. 2013). Each layer describes the different types of interactions. An example of a multiplex network is a social network in which the different layers represent different types of social relationships. For instance, we can assign friendship ties, family ties, and co-worker ties in three different layers.

**Fig. 1:**
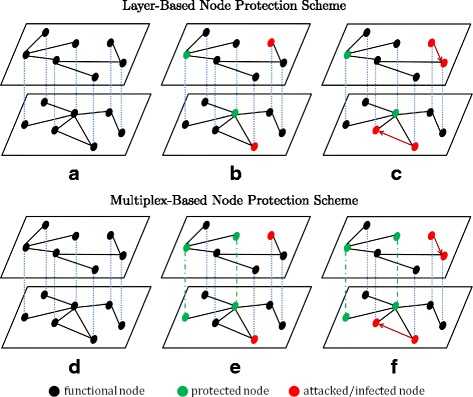
Schematic Illustration of Different Protection Scheme. **a** Input graph. **b** Initial epidemics and protection condition at timestamp *t*=0. **c** Infected nodes start to infect their neighbors at timestamp *t*=1. **d** Input graph. **e** Initial epidemics and protection condition at timestamp *t*=0. **f** Infected nodes start to infect their neighbors at timestamp *t*=1

Capturing the role of multiplex topologies to understand the dynamic of complex networks is still a challenging task (Wu et al. 2016). Given a multiplex graph, such as social network, and *k* budget of protection resources, we aim to protect a set of nodes such that the percentage of survived nodes at the end of epidemics is maximized. If protection is given to a certain node *v* in graph *G*, then *v* could not be infected by its neighbors at any timestamp during epidemic. Specifically, all corresponding edges of *v* in *G* are removed, which means *v* is effectively isolated during epidemic. The epidemic spreading in a multiplex network can occur throughout the connection of all corresponding layer. Thus, the protection scheme in multiplex networks can be classified into two basic classes: multiplex-based and layer-based node protection schemes. Figure 1 illustrates this classification (see “Problem formulations” section for a detailed protection scheme classification).

**Fig. 2:**
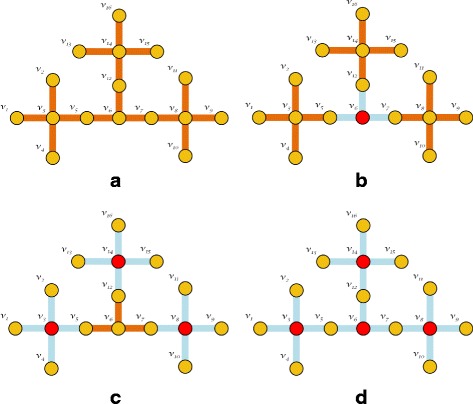
Schematic Illustration of Finding *Centers* and *Bridges* in Graph. The red dots represent the protected nodes. Shaded lines represent the removed edges. **a** Initial Input Graph. **b** Protecting the *Bridges*, nodes with highest value of Random Walk Normalized Fiedler Vector (*μ*). **c** Protecting the *Centers*, nodes with highest value of Degree Centrality (*d*). **d** Combining (b) and (c) objectives in MultiplexShield

The goal of our work is to develop an effective and efficient method that is scalable for protecting multiplex networks. Firstly, we allocate a novel nodes importance ranking score which combines the benefit of algebraic connectivity and degree centrality of graph structure. Intuitively, using those two benefits, we can define both of the connectivity and the centrality role of a certain node. Thus, under *k* limited budget, we can select a set that consists of *k* nodes which have the role as *bridges* and *centers* of the graph. We consider nodes that having the highest degree centrality role as *centers*. We also assume nodes with the highest value of connectivity, measured by random walk normalized Fiedler vector (von Luxburg 2007), as *bridges*. This idea is depicted in Fig. 2 (see “MULTIPLEXSHIELD: pre-Emptive spectral graph protection” section for our detailed proposal). To this end, we got the most suitable nodes to be protected. This node score consists of the corresponding random walk normalized Fiedler vector of nodes in graph, degree value, and layer-wise epidemic stochastic propagation rate. We use SIS model in our work. In SIS propagation model (Gray et al. 2011), we calculate the stochastic propagation rate from the ratio between the infection probability of one node to infect its neighbor and the recovery probability of infected node. This rate represents the strength of propagation and exhibits how quick the epidemics will spread. Prakash et al. (2011) showed that the strength of propagation in SIS model, as well as in SIR, SIRS, and SEIR model depends on this rate.

The main contributions of our paper can be summarized as the following three points:

### Problem formulations

We formalize the problem of suppressing propagation spreading in multiplex network by allocating protection resources throughout the different network layers. We define the Multiplex Graph Protection Problem as a discrete combinatorial optimization. We also introduce that the problem is NP-Hard. To the best of our knowledge, we are the first to analyze the hardness of this multiplex graph protection problem. Furthermore, we also specify two different protection scheme to consider different epidemic spreading scenario in multiplex networks.

### Effective and scalable algorithm

We develop an effective and scalable algorithm to suppress the epidemics spreading on multiplex networks, called MULTIPLEXSHIELD. We find that MULTIPLEXSHIELD is scalable for large graphs and gives more effective protection compared with other competing methods such as Acquaintance Vaccination (AV) (Wang et al. 2015), Targeted Immunization Method (TIM) (Buono and Braunstein 2015), SpreadingDegree (Zhao et al. 2014) and Random Immunization (Zuzek et al. 2015; Wu et al. 2016; Zhao et al. 2014). In addition, we also show the analysis of our proposal, including the complexity of memory allocation and computational complexity. To the best of our knowledge, we are also the first to develop multiplex graph protection strategy by considering and evaluating not only the effectiveness but also the scalability of method for large size graph application.

### Extensive evaluations

We perform comprehensive experiments on multiple real-world network datasets. Our proposed algorithms outperform other competing methods. We also show that MULTIPLEXSHIELD is scalable with respect to the changing of graph size in terms of number of nodes and edges, which means it is suitable for large size graphs.

The remainder of this paper is organized in the following manner: We review the recent most related studies in “Related work” section. We formalized the problem and definition in Problem Formulation section. We present and analyze our proposed methods in “MULTIPLEXSHIELD: pre-Emptive spectral graph protection” section. The result of experimental simulations are provided in Evaluations section. Finally, we elaborate the limitation and possible future challenges of our work in the “Discussions” section and provide concluding remarks in “Conclusions”.

## Related work

In this section, we review the related work, which can be categorized into three parts: graph protection, influence maximization, and influence blocking maximization.

### Graph protection

Most of the recent work in graph protection focused on the single-layered graph and does not provide much consideration on multiplex topologies. In single-layered graph protection scheme, there are two common approaches: pre-emptive and post-emptive protection. Two pre-emptive algorithms have been proposed, called NetShield (Tong et al. 2010) and Netshield+ (Chen et al. 2016) which employ the properties of matrix perturbation to find a set of nodes to be immunized (Tong et al. 2010). Later, in 2016, Chen et al. improved the batching strategy of NetShield and demonstrated a better performance using Netshield+ (Chen et al. 2016). In 2017, GraphShield method was proposed by taking into account the role of infection flow, graph connectivity, and outdegree centrality (Wijayanto and Murata 2017). Meanwhile, some approaches to post-emptive graph protection also have been proposed in (Zhang and Prakash 2014; 2015; Song et al. 2015). Zhang and Prakash (2014; 2015) introduced DAVA and DAVA-fast, two polynomial-time heuristics algorithms. NIIP (Song et al. 2015) extracts a maximum directed acyclic graph from the graph then performs a Monte Carlo simulation to estimate the distribution of *k* over each time point *t* given the probability of a healthy node being infected. Nevertheless, compared to these simplified single-layered isolated assumptions, the multi-layered approach is more realistic due to the common interconnected properties of human social network.

The most related work to ours is multiplex graph protection. Zuzek et al. investigated random immunization method to protect *k* random functional nodes (Zuzek et al. 2015; Wu et al. 2016; Zhao et al. 2014). Wang et al. proposed acquaintance method which selects a set of random neighbor of a randomly chosen node (Wang et al. 2015). Later, Buono *et al* employed top *k* high-degree nodes for protection, termed Targeted Immunization Method (TIM) (Buono and Braunstein 2015). Similarly, Zhao et al. introduced an improvement of TIM, called Spreading Degree (Zhao et al. 2014). More recently, two independent work investigated several protection methods and concluded in favor of Explosive Immunization (EI) and Simulated Annealing (SA) methods (Osat et al. 2017*;*Zhao et al. 2017). The former method removed all the vertices the gradually reinserted to the network but aiming to prevent the formation of the giant connected component (GCC), until a stage where the GCC formation is inevitable. To add a new node, each node is measured and then chosen by a predefined kernel function. A major disadvantage of this method is that its total iteration get computationally costly, especially for large size network (Osat et al. 2017). The later method reintroduced one type of a traditional metaheuristic, which unfortunately not scalable and required a significant amount of running time to converge (Nourani and Andresen 1998*;*Du and Swamy 2016). Thus, both of EI and SA are not promising for large size network application.

All of these works on multiplex graph protection assumed that the infection would start from a random node, which called random attack. In contrast, our work enhances this assumption by also investigating the more powerful type of attack, targeted high-degree attack. Also, contrary to previous approaches which accomplish protection strategy without scalability objective, we aim to develop a more effective and faster method that scales to large size network. Furthermore, the elaboration of spectral properties of the graph as our proposal has not been considered in these recent literatures.

### Influence maximization

The influence maximization task in multi-layered topology shares a similar goal with ours. It aims to find a set of vertices to control the influence propagation in the network. However, while the influence maximization task aims to maximize the influence spreading (Nguyen et al. 2013*;*Zhang et al. 2016*;*Zhang and Zhang 2015), the graph protection tries to encounter and limit those spreading process. Nguyen et al. (2013) demonstrated a coupling scheme to reduce the multiplex graphs into a single layer graph by maintaining the influence properties, therefore applying influence maximization task in the reduced network. Despite the benefit of that lossless coupling scheme, Zhang et al. (2016) introduced a lossy coupling scheme of multiplex influence maximization to overcome the running time and memory consumption issues.

### Influence blocking maximization

He et al. (2012) introduced the influence blocking maximization (IBM) problem to elaborate the competitive influence propagation in social networks under the competitive linear threshold (CLT) model. In IBM problem, one entity aims to block the influence propagation of its opposing counterpart as much as possible by strategically selecting a set of seed nodes to initialize its own influence. IBM problem is another type of competitive influence maximization under the constraint of opposing effect of each party’s influence. For instance, when a negative rumor spreads in the social network about an institution, the institution needs to respond quickly by choosing other seed nodes to inject positive opinions about the institution. The positive opinions spreading are expected to fight against the negative rumor. Thus, in IBM problem, the positive opinions also spread over the network with a certain infection probability. While in our graph protection problem, each protected set of nodes are selected pre-emptively and have no probability to transmit the protection attributes to their neighbors.

To summarize, none of these literatures focused on the study of suppressing the epidemic spreading via pre-emptive spectral graph protection in multiplex networks.

## Problem formulation

In this section, we will formalize the definitions and problems used throughout this paper and describe the classification of protection scheme in multiplex networks. We summarize the terms and notations in Table 1. With the above terms and notations, we can formally describe definitions and problems as follows:

**Table 1:** Summary of terms and notations

Notation	Definition and description
*G*=(*V*,*E*_1_,…,*E*_*L*_)	Multiplex graph *G* with the node set *V* and the edge set *E*_1,…,*L*_
*A*	Multiplex supra adjacency matrix of graph *G*
$\mathcal {L}(A)$	Combinatorial Laplacian matrix of *A*
$\mathcal {L}_{sym}(A)$	Symmetric normalized Laplacian matrix of *A*
$\mathcal {L}_{rw}(A)$	Random walk normalized Laplacian matrix of *A*
*n*	Number of nodes in each multiplex layer
*m*	Number of edges in each multiplex layer
*N*	Number of nodes in graph *G*
*M*	Number of edges in graph *G*
*L*	Number of layers in graph *G*
*d*(*i*)	Degree value (or outdegree value in directed graph) of node *i*
*PV*(*i*)	Protection Value of node *i*
*α*	Algebraic connectivity of $\mathcal {L}_{rw}$
*μ*(*i*)	Corresponding Fiedler vector of $\mathcal {L}_{rw}$ for node *i*
*β*(*i*)	Infection probability at layer *i*
*δ*(*i*)	Recovery probability at layer *i*
*ϕ*	Number of initial infected nodes in a graph
*k*	Number of available protection resources
*S*	Set of nodes selected for protection
*η*_*G*_(*S*)	Number of survived nodes of graph *G* at the end of epidemics
*θ*_*G*_(*S*)	Percentage of survived nodes of graph *G* at the end of epidemics
*θ*_*ave*_	Average of *θ*_*G*_(*S*)
*θ*_*std*_	Standard deviation of *θ*_*G*_(*S*)

### **Definition 1**

Multiplex network is denoted by *G*=(*V*,*E*_1_,…,*E*_*L*_) where *V*={*v*_1_,…,*v*_*n*_} is the node set and *E*_*l*_={*e*_1,*l*_,…,*e*_*m*,*l*_} is the set of edges corresponding to layer *l*. The edge set *E* in a multiplex network is the union of edge sets *E*_*l*_ for *l*={*l*_1_,…,*l*_*L*_}. We can fully describe the structure of *G* by considering the set of adjacency matrices

1 $$ G \equiv A = \{A_{1}, A_{2}, \ldots, A_{L}\},  $$

where *A*_*l*_=*a*_*ij*,*l*_ be the adjacency matrix of layer *l*, with *a*_*ij*,*l*_>0 if node *i* and *j* in layer *l* share a relationship type of *l* and *a*_*ij*,*l*_=0 otherwise. Let consider the coupling matrices or inter-layer adjacency matrices as identity matrix *I*, when all nodes in graph *G* participate in all multiplex layer or commonly defined as the fully-aligned multiplex network (Kivela et al. 2014). Thus, we can also obtain the multiplex supra-adjacency matrix of *G* as

2 $$ A = \left[\begin{array}{cccc} A_{1} & I & \cdots & I \\ I & A_{2} & \cdots & I \\ \vdots & \vdots & \ddots & \vdots \\ I & I & \cdots & A_{L} \\ \end{array}\right] = \bigoplus_{l=1}^{L} A_{l} + I  $$

To maintain consistency and brevity, unless specified otherwise, we define *G* as the undirected network for all proofs and explanations in this paper. However, the generalization to the directed case can be performed without difficulty.

### **Definition 2**

**Susceptible-infected-susceptible (SIS) propagation model**. SIS model defined that each node in graph *G*=(*V*,*E*) with *N* number of nodes, would be in one of the following two states: *susceptible* and *infected*. Let $\mathcal {S}(t)$ be the number of susceptible nodes, and let $\mathcal {I}(t)$ be the number of infected individuals at time *t*. At each timestamp *t*, susceptible nodes can be infected by their infected neighbors with probability *β*. Also, each infected node can get recovered to susceptible state with recovery probability *δ*. This model can be formalized as nonlinear differential equations:

3 $$ \frac{ds}{dt} = -\beta i s, \frac{di}{dt} = \beta i s - \delta i,  $$

being $s(t) = \mathcal {S}(t)/N$ and $i(t) = \mathcal {I}(t)/N$ the respective proportions of states at time *t*.

### **Definition 3**

**Multiplex graph protection problem**. The input is given as follows: an undirected multiplex graph *G*=(*V*,*E*_1_,…,*E*_*L*_) with node set *V* and edge set *E*, SIS propagation model with infection probability *β* and recovery probability *δ* and an integer budget of *k* protection. Let us denote *S*, a subset of *k* nodes from graph *G* selected for protection. We define *θ*_*G*_(*S*) to be the percentage of survived nodes of graph *G* at the end of epidemics given that *S* was protected. Our goal is to find *S*∈*V* such that *θ*_*G*_(*S*) is maximized, subject to the size of *S* is equal to constraint *k*, i.e. calculating the following discrete combinatorial optimization:

4 $$ \begin{aligned} S^{*} &= \underset{S \in V}{ \text{argmax}}\ \theta_{G}(S) \\ \text{s.t.} |S| &= k \\ \end{aligned}  $$

### **Theorem 1**

Multiplex graph protection problem is NP-Hard.

### *Proof*

Zhang and Prakash (2014) have presented that Data-Aware Vaccination (DAV) problem is NP-Hard by reducing Minimum K-Union (MinKU) set problem (Vinterbo 2004) which was proven to be hard. In Wijayanto and Murata (2017), the authors proved the special case of MinKU, called FAVP, to be hard. They reduced the MinKU problem to an instance of FAVP problem with *δ*=1 and *β*=1, given that MinKU has instance a set *S* where *S*_*i*_⊆*V* and positive integer *k*. The Multiplex Graph Protection problem can be derived as a generalization of FAVP problem where *L*>1 for any given *δ* and *β*.

In the minimum *k*-union (MinKU) problem, we are given a set system with *s* sets and have to select *k* sets to minimize the size of their union. As in MinKU problem there is no specification of changing the state of each set as well as no transmission of state be specified, then we can reduce MinKU to an instance of multiplex graph protection problem in SIS model with *β*=1, *δ*=1, and the number of layer *L*=1. We can specify that this MinKU problem as a special case of multiplex graph protection problem. However, in the multiplex graph protection problem, the value of *β* and *δ* may vary as well as the number of layer *L*>1. While MinKU problem has proven to be NP-Hard (Vinterbo 2004), we can demonstrate that multiplex graph protection problem under SIS propagation model with any values of *β* and *δ* and consists of multiple layers is also NP-Hard. □

### Protection scheme

Here we will specify the classification of protection scheme in multiplex networks. Recall that due to the interconnected properties of a multiplex network, the epidemic spreading can occur along the link of all corresponding layer of the network. Hence, the node protection scheme can be classified into two basic classes:

- *Layer-based Node Protection Scheme*, where each protected node is uninfectable by its neighbors in a certain layer and meanwhile, its corresponding nodes in other layers still can get infected by their neighbors. This scheme can approximate the situation when the protection means an isolated state from infection spreading (Wu et al. 2016*;*Zhao et al. 2014). Thus, the isolation is only applicable in such layer. For instance, in a multiplex social network of neighborhood and colleague relationship, a person who is isolated from his/her office will lose connections to all of his/her colleagues, but still likely keeps connected with his/her neighbors. Thus the person is still infectable by his/her neighbors. In computer virus spreading, the office may provide personalized proxy or firewall for some of their employees’ notebook under WiFi connection. However, his/her notebook may still got infected at home or somewhere else. Another example, for a certain type disease without any proven vaccination, one student may got instructed to wear face mask at school. But, he/she may still got the disease from his/her neighbors.
- *Multiplex-based Node Protection Scheme*, where each protected node is uninfectable during the contamination spreading in all corresponding layers. This scheme is most suitable for multilayer graph where each individual nodes are in the same certain awareness (Wu et al. 2016*;*Zhao et al. 2014). Thus, giving a protection to a certain node will change its states of corresponding nodes in all layers.

We illustrated these protection schemes in Fig. 1. In Fig. 1a, we are given a multiplex graph as input for Layer-based Node Protection Scheme. Let the number of available protection resources *k*=2 and the number of initial infected nodes *ϕ*=2 which are equally allocated at each layer, we got the current epidemics spreading and protection at timestamp *t*=0 as illustrated in Fig. 1b. Since it is layer-based node protection scheme, all of corresponding nodes of the protected nodes in other layers are still remain functional, i.e. not protected. Then, each of infected nodes has *β* probability of infecting its neighbors. Fig. 1c shows the example of current epidemics at timestamp *t*=1.

On the other hand, we provide the schematic illustration of the Multiplex-based Node Protection Scheme in Fig. 1d-f. For comparability, we are also given the same multiplex graph as input, as shown in Fig 1d. Additionally, we also use the same setting of *k* and *ϕ* to be equally allocated at each layer. As illustrated in Fig. 1e, each protected node is uninfectable during the contamination spreading in all corresponding layers, which give more protection benefit.

In this paper, we consider and investigate both of these protection schemes.

## MULTIPLEXSHIELD: pre-Emptive spectral graph protection

In this section, we will describe our proposed pre-emptive spectral graph protection and justify our approach. Recall that our goal is to develop an effective and efficient method that is scalable for protecting multiplex networks. We introduce a novel nodes importance ranking score as a basis for determining *k* set of protected nodes *S*. Specifically, this ranking score intent to quantify the importance of set *S*, and the impact of their protection to the rest of the graph. With consideration of the spectral properties of multiplex topologies and epidemic propagation rate, we introduce this ranking based on three objectives.

*Firstly*, we aim to determine the nodes having the role as *bridges* connecting sub structures or spectral clusters in graph. Inspired by the benefit of algebraic connectivity of graph, graph partitioning task and spectral clustering problem, we propose the random walk normalized Fiedler vector to find such nodes in multiplex graph.

*Secondly*, we aim to find the nodes have the *centers* role in multiplex graph. We assume that this role can be determined based on the highest degree centrality value of nodes.

*Thirdly*, we aim to anticipate the different epidemic propagation rate in multiplex topologies. We propose to calculate the layer-wise stochastic propagation rate from the ratio between the infection probability of one node to infect its neighbor and the recovery probability of infected node. This rate represents the strength of propagation and exhibits how quick the epidemics will spread.

Figure 2 illustrates the simplified example of schematic representation to implement our first and second objectives. Let an initial epidemic graph in Fig. 2a is given as input, which we may assume as a subset of a layer in an arbitrary multiplex graph. Intuitively, we want to localize any incoming epidemic spreading in the future by disconnecting spectral clusters of graph. In Fig. 2b, we select node *v*_6_, which has the highest value of the random walk normalized Fiedler vector, to be protected. Specifically, all corresponding edges of *v*_6_ in graph are removed. This means *v*_6_ is effectively isolated during epidemic. Thus, also localized the future epidemic spreading into three disconnected clusters. Then, in Fig. 2c, we aim to protect the *centers* by selecting the nodes with highest value of degree centrality. Subsequently, we combine the Fig. 2b-c objectives to protect the whole given graph.

Next we will describe the key components of our proposed method and clarify the justification in more detail.

### Protecting *bridges*: random walk normalized Fiedler vector

To determine most suitable nodes for protection, we can intuitively localize any epidemic spreading by disconnecting sub-structures or clusters of the network. Motivated by the insight in graph partitioning and spectral clustering (Brouwer and Haemers 2012*;*von Luxburg et al. 2008), we introduce random walk normalized Fiedler vector to obtain the nodes having the role as *bridges* in a graph.

Let *G*=(*V*,*E*) be an undirected graph with non-negative weights, *N* number of nodes and an adjacency matrix *A*. We can denote the degree matrix *D* as the diagonal matrix with the degree values *d*_1_,…,*d*_*N*_ on the diagonal, *I* as the identity matrix and define the combinatorial Laplacian matrix as $\mathcal {L} = D - A $. We order the eigenvalues of $\mathcal {L}$ so that 0=*λ*_1_≤*λ*_2_≤*λ*_3_≤…≤*λ*_*N*_ with corresponding mutually orthonormal eigenvectors *v*_1_,*v*_2_,…,*v*_*N*_. We refer to *λ*_2_ and *v*_2_ as the algebraic connectivity and the Fiedler vector of the Laplacian, respectively.

Assuming that *D* is invertible, we can define two different normalized Laplacian matrices: the symmetric normalized Laplacian matrix $\mathcal {L}_{sym}$ and the random walk normalized Laplacian matrix $\mathcal {L}_{rw}$, as follow:

5 $$ \mathcal{L}_{sym} := D^{-1/2} \mathcal{L} D^{-1/2} = I - D^{-1/2} A D^{-1/2}  $$

6 $$ \mathcal{L}_{rw} := D^{-1} \mathcal{L} = I - D^{-1} A  $$

We denote the symmetric normalized Laplacian matrix $\mathcal {L}_{sym}$ as it is a symmetric matrix, and the random walk normalized Laplacian matrix $\mathcal {L}_{rw}$ as it is associated with the transition matrix of stochastic graph random walk, *P*=*D*^−1^*A*.

The $\mathcal {L}_{rw}$ has the non-negative real-valued eigenvalues which can be ordered so that 0=*α*_1_≤*α*_2_≤*α*_3_≤…≤*α*_*N*_ with corresponding mutually orthonormal eigenvectors *μ*_1_,*μ*_2_,…,*μ*_*N*_. We refer to *α*_2_ and *μ*_2_ as the random walk normalized algebraic connectivity and the random walk normalized Fiedler vector, respectively. For simplicity, we will use the notation *α* and *μ* to denote *α*_2_ and *μ*_2_.

The multiplicity *ω* of the eigenvalue 0 of both $\mathcal {L}_{sym}$ and $\mathcal {L}_{rw}$ are equivalent to the number of connected components in graph (von Luxburg 2007). While, the Fiedler vector of all $\mathcal {L}, \mathcal {L}_{sym}$ and $\mathcal {L}_{rw}$ can be used to separate graph *G* (Driessche and Roose 1995*;*von Luxburg et al. 2008), in the sense of selecting separator among clusters or subgraphs. An essential question then arises, which of the three Laplacian matrices should be used to compute the Fiedler vector? We clarify our proposal of involving the random walk normalized Fiedler vector as follows:

#### 1. Graph partition point of view

In graph partitioning task, a graph *G*=(*V*,*E*) can be partitioned into two disjoint subsets, *X*,*Y*, where *X*∪*Y*=*V*,*X*∩*Y*=*∅*, by removing edges connecting the two subsets. A straightforward approach to construct this partition is by solving the *mincut* problem. Let denote the weighted adjacency matrix of the graph is the matrix *W*. In the *mincut* problem, we denote $W(X,Y):= {\sum \nolimits }_{i \in X, j \in Y} w_{ij}$, where *w*_*ij*_ is the weight of edges connecting node *i* and *j*. We also denote the complement of a subset *X*⊂*V* as $\bar {X}$. The *mincut* problem aims to choose partitions *X*_1_,…,*X*_*k*_ for a given number *k* of subsets which minimizes

7 $$  \text{cut}(X_{1}, \ldots, X_{k}) := \frac{1}{2} \sum\limits_{i = 1}^{k} W(X_{i}, \bar{X}_{i})  $$

Even though the *mincut* problem is obviously solvable, it does not guarantee satisfactory partitions. In many practical cases, the solution simply separates a single node from the rest of the graph. To overcome this issue, two common objectives functions had been proposed to improve the partition quality: RatioCut and NCut. Given that |*X*| denote the size of a subset *X* measured by its number of nodes, we can also denote vol(*X*) as the weights of all edges in *X*. These objective functions aim to minimize:

8 $$  \text{RatioCut}(X_{1}, \ldots, X_{k}) := \frac{1}{2} \sum\limits_{i = 1}^{k} \frac{W(X_{i}, \bar{X}_{i})}{|X_{i}|} = \sum\limits_{i = 1}^{k} \frac{\text{cut}(X_{i}, \bar{X}_{i})}{|X_{i}|}  $$

9 $$  \text{NCut}(X_{1}, \ldots, X_{k}) := \frac{1}{2} \sum\limits_{i = 1}^{k} \frac{W(X_{i}, \bar{X}_{i})}{\text{vol}(X_{i})} = \sum\limits_{i = 1}^{k} \frac{\text{cut}(X_{i}, \bar{X}_{i})}{\text{vol}(X_{i})}  $$

Using the Rayleigh-Ritz theorem, the solution of RatioCut minimization can be approximated by the Fiedler vector of $\mathcal {L}$ unnormalized Laplacian of graph *G*. And by applying the similar approximation to NCut minimization, we can achieve both the Fiedler vector of $\mathcal {L}_{sym}$ symmetric normalized and $\mathcal {L}_{rw}$ random normalized Laplacian of graph *G* (von Luxburg 2007).

Recall that in Eq. 8, the minimization of RatioCut which employs unnormalized Laplacian can specify partitions such that nodes in the different cluster are dissimilar to each other. This means that RatioCut can minimize the similarities between clusters. While the NCut, as stated in Eq. 9, which employ the normalized Laplacian not only can achieve the same objective as RatioCut, but also able to maximize the similarities within clusters. The best partition of graph has low similarities between clusters and high similarities within clusters. To this end, $\mathcal {L}_{rw}$ and $\mathcal {L}_{sym}$ are in favor as the Fiedler vector base, than $\mathcal {L}$.

#### 2. Statistical consistency

Looking at the statistical consistency differences between the two normalized Fiedler vector of $\mathcal {L}_{rw}$ and $\mathcal {L}_{sym}$, von Luxburg (2007*); von Luxburg et al. (*2008) investigated the spectral clustering algorithms results of $\mathcal {L}_{rw}$ and $\mathcal {L}_{sym}$. The eigenvectors of $\mathcal {L}_{rw}$ are cluster indicator vectors $\mathbb {1}_{A_{i}}$. However, the eigenvectors of $\mathcal {L}_{sym}$ are additionally multiplied with *D*^1/2^, which might lead to undesired convergences. Empirical results of spectral clustering are also in favor of $\mathcal {L}_{rw}$. As using $\mathcal {L}_{sym}$ does not have any computational benefits, selecting $\mathcal {L}_{rw}$ is more preferable.

#### 3. Regular and irregular graph applicability

Let us consider the degree distribution of the similarity graph. The utilization of Laplacian matrix $\mathcal {L}$ can provide a satisfactory result for partitioning the regular graphs. If the graph *G*=(*V*,*E*) is very regular and most nodes have approximately the same degree, then all the Laplacians $\mathcal {L}$, $\mathcal {L}_{rw}$ and $\mathcal {L}_{sym}$ will deliver similar and consistent result. In contrary, if the degree distribution of graph *G* are very broadly distributed, the partitions of using Fiedler vector of $\mathcal {L}$ are considerably worse than that of $\mathcal {L}_{rw}$. Therefore, the random walk normalized Laplacian $\mathcal {L}_{rw}$ are applicable in irregular graphs as well as in regular graphs (von Luxburg 2007).

### Protecting *centers*: degree centrality

The role of degree centrality in networks has been discussed by some recent studies (Solé-Ribalta et al. 2014*;*Buono and Braunstein 2015*;*Zhao et al. 2014). There are many benefits of prioritizing the high degree nodes among others. In this work, we incorporate the benefit of this centralization to our method. Regard to the directed multiplex networks, the outdegree centrality is preferable. Outdegree centrality counts the number of neighbors that a certain node can infect.

### Layer-wise epidemic stochastic propagation rate

Given an arbitrary epidemic network, nodes can possess different states depending on the epidemic model. The model we simulate in this work is SIS model. In the SIS model, each node would belong to either susceptible or infected state. Susceptible nodes can be infected by their neighbors with infection probability *β* at each time stamp, and each infected node can recover to susceptible state with recovery probability *δ*.

In multiplex networks, different infections spread along different layer with specific stochastic propagation rate. Intuitively, this is the speed of spreading infection. In our work, we consider $\frac {\beta (i)}{\delta (i)}$ as stochastic propagation rate at layer *i*.

#### **Lemma 1**

In multiplex network, stochastic propagation rate of layer-wise epidemic spreading is defined by $\frac {\beta }{\delta }$

#### *Proof*

We consider a multiplex network *G*=(*V*,*E*_1_,…,*E*_*L*_) with *L* number of layer. Let us recall the nonlinear differential equations of SIS, $\frac {ds}{dt} = -\beta i s, \frac {di}{dt} = \beta i s - \delta i$. In any particular layer *l*, given that *s*+*i*=1, we can reformulate *s* as a function of *i* as follows:

10 $$ \frac{di}{dt} = \beta i(1-i) -\delta i = (\beta - \delta)i - \beta i^{2}  $$

This is an instance of a logistic equation. We can show that $\frac {\beta }{\delta }$ determine *i*(*t*) using this logistic equation. If *i*(0)>0, it can be inferred that when $\frac {\beta }{\delta } \leq 1$, then ${\lim }_{t \to + \infty } i(t) = 0$. If $\frac {\beta }{\delta }>1$, then ${\lim }_{t \to + \infty } i(t) = 1 - \frac {\delta }{\beta }$. □

Prakash et al. also demonstrated in empirical simulations that the $\frac {\delta }{\beta }$ takes the role as constant dependent of epidemic threshold not only in SIS, but also in SIR, SIRS, and SEIR model (Prakash et al. 2011). Epidemic threshold is an intrinsic property of a network. When the strength of the virus is greater than the epidemic threshold, then the epidemic would breakout (Prakash et al. 2011).


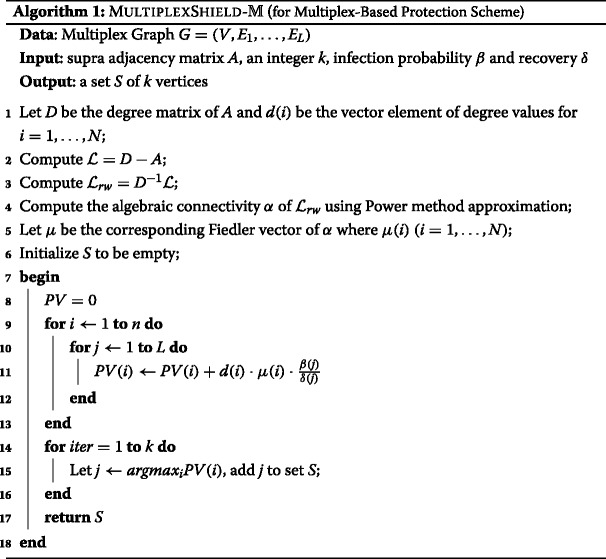


### MultiplexShield algorithms

Let us recall our goal to develop an effective and efficient method that scalable for protecting multiplex networks. Here we will describe our proposal, MULTIPLEXSHIELD algorithms. We define a novel nodes importance ranking score, called Protection Value (PV), as a basis for determining *k* set of nodes *S* to be pre-emptively protected. The higher score of the ranking, the higher importance of nodes to be selected under MULTIPLEXSHIELD. Protection Value is composed by considering the three previously explained objectives: protecting *bridges* of graph by random walk normalized Fiedler vector value; protecting *centers* of graph by degree centrality value; and layer-wise epidemic propagation rate.

In addition, to distinguish two classifications of protection scheme in multiplex networks, we consider two implementation versions. The $\textsc {MultiplexShield-}\mathbb {M}$ Algorithm is specified for multiplex-based protection scheme. We calculate the Protection Value of node *i* under assumption that the benefit of finding *bridges* and *centers* in the graph are equally important. Therefore, given a multiplex network *G* with *L* number of layer, the PV of nodes *i* is given by:

11 $$ PV(i) = \sum\limits_{j=1}^{L} d(i) \cdot \mu(i) \cdot \frac{\beta(j)}{\delta(j)},  $$

being *β*(*j*) and *δ*(*j*) the respective infection probability and recovery probability at layer *j*, while *μ*(*i*) is the *i*-th element of *μ* vector.

*μ* vector consists of *n* elements at size of the number of nodes in the graph. The corresponding eigenvector of second smallest Laplacian of the graph dictates the optimal partition of the graph, which each element determine each node belong to a certain subpartition (Shi and Malik^2000^*; Ng et al.*^2001^). Using the nature of *μ* vector which approximates the minimization of normalized cut (Ncut), it forces nodes to create natural subpartition of graph based on *μ*(*i*) value (Shi and Malik 2000).

The more detail of $\textsc {MultiplexShield-}\mathbb {M}$ is given in Algorithm 1. It requires the adjacency matrix *A* and an integer *k* as the input and provides a set *S* of *k* vertices as the output. We compute the random walk normalized Fiedler vector in step 4-5. The positive and negative values of the random walk normalized Fiedler vector are treated equally. Then we initialize empty set *S* in step 6. The *N*x1 vector PV measures Protection Value of each individual node. Then, in each iteration of steps 14-16, we select top *k* nodes and add it into set *S* according to PV (step 11).

Furthermore, our implementation versions of layer-based protection scheme is $\textsc {MultiplexShield-}\mathbb {L}$ Algorithm. Assuming that the graph *G* has *n* number of nodes in each multiplex layer, we calculate layer-wise PV of node *i* in layer *j* as:

12 $$ PV(i + n \cdot j) = d(i + n \cdot j) \cdot \mu(i + n \cdot j) \cdot \frac{\beta(j)}{\delta(j)}  $$

Algorithm 2 explains the detailed procedure of $\textsc {MultiplexShield-}\mathbb {L}$. Given the adjacency matrix *A* and an integer *k* as the input, it results a set *S* of *k* nodes.


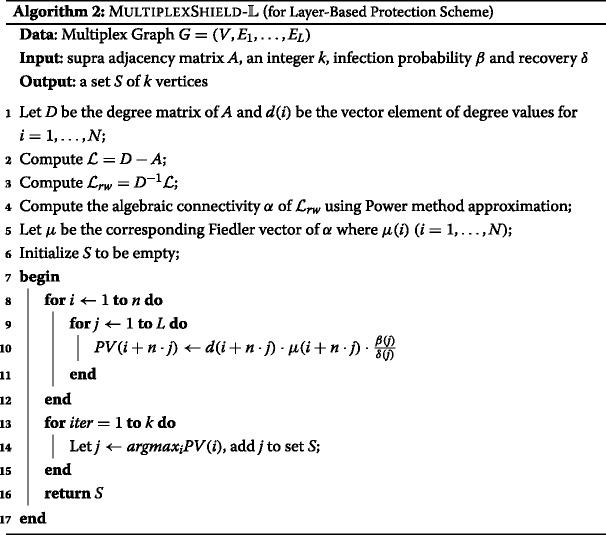


Next, we will provide the analysis of MULTIPLEXSHIELD algorithm in terms of computational complexity and cost of space.

### Computational time complexity analysis

We will analyze first the computational time complexity of $\textsc {MultiplexShield-}\mathbb {M}$ algorithm (for Multiplex-Based Protection Scheme). In Algorithm 1, the cost of calculating the second smallest eigenvalue of random walk normalized Laplacian (step 4) is *O*((*N*+*M*).(log*N*)^*O*(1)^) using the Power method approximation (Trevisan 2014). If the graph is sparse and approximation error threshold (*ε*) is defined small, then the time needed will be almost linear, $O\left (\frac {1}{\epsilon } m \log n \right)$. We know that the cost of step 1,3,5, and 6 are constant. Steps 8-13 cost *O*(*n*). For steps 14-16, its cost is *O*(*k*).

13 $$  \begin{aligned} cost(\textsc{MultiplexShield-}\mathbb{M}) & = O\left(\frac{1}{\epsilon} M \log N\right) + O(N) + O(k)\\ & = O\left(\left(\frac{1}{\epsilon} M \log N\right)+(N +k)\right) \end{aligned}  $$

Consequently, akin to Algorithm 1, we can also infer the computational time complexity of Algorithm 2

14 $$  cost(\textsc{MultiplexShield-}\mathbb{L}) = O\left(\left(\frac{1}{\epsilon} M \log N\right)+(N +k)\right)  $$

### Memory allocation complexity analysis

The required memory allocation or space cost of steps 1-5 in Algorithm 1 are *O*(*N*+*M*+1):*O*(*M*) for storing the graph, *O*(*M*) for storing the degree matrix, *O*(*N*+*M*) for running the eigen-decomposition algorithm, *O*(1) for storing *α*, *O*(*N*) for storing *μ*, and *O*(*N*) for storing the degree (*d*). The cost for step 6 is *O*(1). The space cost of steps 8-13 is *O*(*N*) which re-usable during the iteration. Lastly, to store the output *S* set of nodes, we need *O*(*k*). By ignoring the constant term, we can summarize that the space cost of Algorithm 1

15 $$  space(\textsc{MultiplexShield-}\mathbb{M}) = O(N + M + k)  $$

Similarly, we can also infer the space cost of Algorithm 2

16 $$  space(\textsc{MultiplexShield-}\mathbb{L}) = O(N + M + k)  $$

## Evaluations

In this section, we will provide experimental evaluation of MULTIPLEXSHIELD. The goal of this evaluation was to answer the following questions:

1. *(Effectiveness)* How effective is the proposed MULTIPLEXSHIELD in suppressing propagation spreading of real multiplex graphs? We define the measurement of effectiveness using the percentage of survived nodes of graph at the end of epidemics (*θ*_*G*_(*S*)).

2. *(Scalability)* How scalable is the proposed MULTIPLEXSHIELD with respect to the changing of graph size (*n* and *m*) and different *k* budget size?

### Datasets

We run our experiments on various real multiplex network datasets, which summarized in Table 2.

**Table 2:** Statistics of dataset

Name	#Layers	#Nodes	#Edges	Density	Type
Florentine Families	2	16	35	0.0726	undirected
Krackhardt HighTech	3	21	312	0.1470	directed
Vicker 7thGrader	3	29	740	0.1211	directed
Lazega LawFirm	3	71	2,223	0.0589	directed
Physician Innovation	3	246	1,551	0.0062	directed
C.Elegans	3	279	5,863	0.0064	undirected
Kapferer TailorShop	4	39	1,018	0.0572	directed
CS Aarhus	5	61	620	0.0223	undirected
*Structure of each layer*					
Name	#Edges of layer		Average degree of layer		
Florentine families	20; 15		1.3333; 1.0000		
Krackhardt HighTech	190; 102; 20		9.0476; 4.8571; 0.9524		
Vicker 7th grader	361; 181; 198		12.4483; 6.2414; 6.8276		
Lazega LawFirm	892; 575; 1104		12.5634; 8.0986; 15.5493		
Physician innovation	480; 565; 506		1.9917; 2.3444; 2.0996		
C.Elegans	1031; 1639; 3193		3.6953; 5.8746; 11.4444		
Kapferer TailorShop	316; 446; 109; 147		8.1026; 11.4359; 2.7949; 3.7692		
CS Aarhus	193; 124; 21; 88; 194		3.1639; 2.0328; 0.3443; 1.4426; 3.1803

- *Florentine Families* consists of 2 layers (marriage alliances and business relationships) describing Florentine families in the Renaissance (Padgett and Ansell 1993).
- *Krackhardt HighTech* is the multiplex social network between managers of a high-tech company consists of 3 kinds of relationships (Advice, Friendship, and “Reports to”) (Krackhardt 1987).
- *Vicker 7thGrader* is the multiplex social network of 29 seventh grade students in a school in Victoria, Australia (Vickers and Chan 1981).
- *Lazega LawFirm* dataset is the multiplex social network consists of 3 kinds of (Co-work, Friendship and Advice) between partners and associates of a corporate law partnership (Lazega 2001).
- *Physicians Innovation* is the dataset representing the multiplex social network of a sample of physicians in 4 US towns: Illinois, Peoria, Bloomington, Quincy, and Galesburg (Coleman et al. 1957).
- *C.Elegans* represents the multiplex neuronal network of the nematode (“Caenorhabditis Elegans”) which consists of 3 layers corresponding to different synaptic junctions: electric (“ElectrJ”), chemical monadic (“MonoSyn”), and polyadic (“PolySyn”) (Chen et al. 2006).
- *Kapferer TailorShop* is the dataset of interactions in a tailor shop in Zambia (then Northern Rhodesia) over a period of ten months. Layers represent two different types of interaction, recorded at two different times (seven months apart) over a period of one month (Kapferer 1972).
- *CS Aarhus* is the multiplex social network consists of five kinds of online and offline relationships (Facebook, Leisure, Work, Co-authorship, Lunch) between the employees of Computer Science department at Aarhus (Magnani et al. 2013).

### Method comparisons

Recall that we consider two different stochastic propagation settings in multiplex protection: multiplex-based and layer-based node protection schemes. Here we compare the performance of the following methods:

- *Random Immunization*: this method gives protection to *k* uniformly random functional nodes. This method was introduced in multiplex-based (Zhao et al. 2014*;*Wu et al. 2016) and layer-based protection scheme (Zuzek et al. 2015)
- *Acquaintance Vaccination (AV)*: this methods picks a set of random neighbor of a randomly chosen node (Wang et al. 2015).
- *Targetted Immunization Strategies (TIM)*: this method chooses *k* based on their degree ranking and combine the corresponding degree values of node in all layer (Buono and Braunstein 2015). Basically, TIM is introduced for multiplex-based protection scheme, but in this evaluation, we also implement the methods for layer-based scheme by selecting top *k* high-degree nodes.
- *Spreading Degree*: which select *k* nodes having the highest multiplication of degree values and transmissibility of epidemics (*β*) in all layer (Zhao et al. 2014). This method can be implemented in multiplex-based and layer-based protection scheme. This is the current state-of-the-art method in multiplex graph protection.
- *MultiplexShield*: our proposed method to select *k* nodes based on spectral properties, degree ranking and stochastic propagation rate in each layer. We use $\textsc {MultiplexShield-}\mathbb {M}$ in multiplex-based protection scheme and $\textsc {MultiplexShield-}\mathbb {M}$ in layer-based protection scheme.

### Evaluation metric

We measure the protection effectiveness result using a percentage of survived nodes of graph at the end of epidemics (*θ*_*G*_(*S*)). We compare the effectiveness of our proposed MULTIPLEXSHIELD methods against the baseline algorithms (Random Immunization, AV, TIM, and SpreadingDegree). On the other hand, we measure the scalability by evaluating the computational time of MULTIPLEXSHIELD on various value of the budget *k* to check how it scales with the changing of graph size (*n* and *m*).

### Effectiveness evaluation

Here we will evaluate the effectiveness of our proposed methods. To comprehensively evaluate and compare methods, we provide simulations for 4 different settings: random nodes attack scenario (both for multiplex-based and layer-based protection scheme) and targeted high-degree nodes attack (both for multiplex-based and layer-based protection scheme as well). In random nodes attack, nodes are picked randomly, while in targeted high-degree nodes attack, the nodes with highest degree values are selected. Note that, common to previous literatures, they only evaluate on random attack scenario and do not consider the targeted attack (Wu et al. 2016*;*Zuzek et al. 2015*;*Buono and Braunstein 2015*;*Zhao et al. 2014). Even the evaluation in single-layered graph protection literatures (Chen et al. 2016*;*Zhang and Prakash 2014*;*Wijayanto and Murata 2017*;*Song et al. 2015*;*Zhang and Prakash 2014), which are more mature, were still limited to random attack.

For the sake of comparability and repeatability, we use the common settings in single-layered graph protection literatures (Chen et al.^2016^*; Zhang and Prakash*^2014^a, b; Wijayanto and Murata^2017^*; Song et al.*^2015^). Number of budget *k*=0.25 of *n*, stochastic infection probability (*β*) and recovery probability (*δ*) were set incrementally in all layers under range 0.5≤*β*≤0.9 similarly 0.5≤*δ*≤0.9 as commonly used by (Chen et al.^2016^*; Zhang and Prakash*^2014^*a, b; Wijayanto and Murata*^2017^*; Song et al.*^2015^). Note that in multilayer network the stochastic probability may be different in all layers. Here we will report the result of average and standard deviation of 100 times simulations equally for all comparison methods.

- *Random nodes attack scenario*
  In random nodes attack scenario, a set of *ϕ* nodes are randomly infected to initialize the epidemics spreading. Refer to previous literatures (Wijayanto and Murata 2017*;*Chen et al. 2016), we also set *ϕ*=*k* to initialize the epidemic process. The result can be described as follows:
  Firstly, in *Multiplex-Based Protection Scheme*, it can be seen from Table 3, that in all datasets MultiplexShield can achieve better performance than other competing methods. Note that the three last methods: TIM, SpreadingDegree, and MultiplexShield can consistently select the same *k* set of nodes in all 100 times simulations. This is due to their consistent selection criteria. On the other hand, Random Immunization and AV, due to their randomness nature, resulted different set of nodes which leads to it worst performance.

**Table 3:** Multiplex-based protection scheme, random nodes attack scenario

Methods	Florentine Families		Krackhardt HighTech		Vicker 7thGrader	
	*θ*_*ave*_	*θ*_*stdev*_	*θ*_*ave*_	*θ*_*stdev*_	*θ*_*ave*_	*θ*_*stdev*_
Random immunization	82.09	5.60	76.94	2.20	77.99	1.89
AV	80.59	2.96	76.81	2.91	78.07	1.30
TIM	86.31	3.74	78.40	1.59	78.60	1.77
SpreadingDegree	86.38	4.86	78.44	1.47	78.56	1.26
MultiplexShield	**88.22**	4.88	**80.11**	2.11	**80.85**	1.25
Methods	Lazega LawFirm		Physician Innovation		C.Elegans	
	*θ*_*ave*_	*θ*_*stdev*_	*θ*_*ave*_	*θ*_*stdev*_	*θ*_*ave*_	*θ*_*stdev*_
Random immunization	77.85	1.01	78.94	0.49	78.90	0.35
AV	77.95	1.04	78.86	0.38	78.96	0.41
TIM	78.51	0.83	79.23	0.38	80.13	0.62
SpreadingDegree	78.51	0.76	79.63	0.32	80.20	0.70
MultiplexShield	**79.43**	0.68	**80.01**	0.43	**81.05**	0.44
Methods	Kapferer TailorShop		CS Aarhus			
	*θ*_*ave*_	*θ*_*stdev*_	*θ*_*ave*_	*θ*_*stdev*_		
Random immunization	80.78	0.72	82.32	0.57		
AV	81.19	0.70	82.48	0.55		
TIM	81.31	0.67	82.71	0.60		
SpreadingDegree	81.42	0.71	82.72	0.55		
MultiplexShield	**82.07**	0.75	**83.21**	0.74
  Secondly, under *Layer-Based Protection Scheme*, our proposed MultiplexShield also outperform other competing methods, as can be inferred from Table 4. Interestingly, we can analyze that under the same settings, multiplex-based protection scheme provides a better environment for all methods to achieve better effectiveness performance than in layer-based scheme.

**Table 4:** Layer-based protection scheme, random nodes attack scenario

Methods	Florentine Families		Krackhardt HighTech		Vicker 7thGrader	
	*θ*_*ave*_	*θ*_*stdev*_	*θ*_*ave*_	*θ*_*stdev*_	*θ*_*ave*_	*θ*_*stdev*_
Random immunization	74.88	2.47	73.49	2.73	73.87	1.49
AV	77.38	3.05	73.92	1.38	74.24	1.29
TIM	85.34	5.79	74.63	1.63	74.18	1.51
SpreadingDegree	85.41	5.12	74.94	1.55	74.26	1.96
MultiplexShield	**87.25**	8.98	**76.25**	2.04	**76.05**	1.33
Methods	Lazega LawFirm		Physician Innovation		C.Elegans	
	*θ*_*ave*_	*θ*_*stdev*_	*θ*_*ave*_	*θ*_*stdev*_	*θ*_*ave*_	*θ*_*stdev*_
Random immunization	73.53	0.80	73.96	0.33	73.97	0.47
AV	74.12	0.74	74.41	0.33	73.81	0.29
TIM	74.27	0.69	74.76	0.46	74.73	0.78
SpreadingDegree	74.49	0.90	74.52	0.70	74.85	0.66
MultiplexShield	**75.15**	0.71	**75.72**	0.56	**75.36**	0.52
Methods	Kapferer TailorShop		CS Aarhus			
	*θ*_*ave*_	*θ*_*stdev*_	*θ*_*ave*_	*θ*_*stdev*_		
Random immunization	75.95	0.84	78.70	0.71		
AV	76.17	1.02	78.69	0.74		
TIM	76.42	0.82	78.82	0.83		
SpreadingDegree	76.46	0.75	78.86	0.70		
MultiplexShield	**77.12**	0.94	**79.33**	0.61
- *Targeted high-degree nodes attack scenario*
  While random attacks are commonly evaluated, we should note that scale-free networks are more vulnerable to high-degree attack due to its power-law degree distribution (Albert et al. 2000). Here we simulate a set of *ϕ*=*k* high-degree nodes as targeted initial infection. Also note that the aforementioned insight in random attack scenario also occurs in this scheme. MultiplexShield gives the consistent selection of *k* set protected nodes. The result of simulation in targeted high-degree attack can be summarized as follows:
  Under *Multiplex-Based Protection Scheme*, Table 5 depicted that our proposed MultiplexShield shows higher effectiveness than other competing methods in almost all of the datasets. In C.Elegans datasets, despite its slightly lower performance compared to degree-oriented methods (TIM and SpreadingDegree), our MultiplexShield is still competitive. The intuition of MultiplexShield result on this dataset under multiplex-based scheme is that the combination of all layer protection value for each node (which covers equally findings of best *centers* and *bridges*) are not suitable. This issue left us future investigation. Instead of equally assigned, what is the most suitable proportion of weighting for *centers* and *bridges* weight for each different graphs in our methods.

**Table 5:** Multiplex-based protection scheme, targeted high-degree nodes attack scenario

Methods	Florentine Families		Krackhardt HighTech		Vicker 7thGrader	
	*θ*_*ave*_	*θ*_*stdev*_	*θ*_*ave*_	*θ*_*stdev*_	*θ*_*ave*_	*θ*_*stdev*_
Random immunization	83.28	3.06	76.86	1.28	77.97	1.18
AV	78.56	2.22	77.46	2.08	78.43	1.22
TIM	87.91	1.06	78.90	1.43	78.46	1.23
SpreadingDegree	88.13	1.26	78.67	1.43	78.62	1.34
MultiplexShield	**89.66**	1.45	**80.25**	1.44	**80.10**	1.35
Methods	Lazega LawFirm		Physician Innovation		C.Elegans	
	*θ*_*ave*_	*θ*_*stdev*_	*θ*_*ave*_	*θ*_*stdev*_	*θ*_*ave*_	*θ*_*stdev*_
Random immunization	78.60	0.81	78.99	0.43	79.42	0.41
AV	78.68	0.85	79.23	0.34	78.82	0.32
TIM	78.85	0.69	79.37	0.45	81.44	0.40
SpreadingDegree	78.93	0.67	79.57	0.52	**81.96**	0.46
MultiplexShield	**79.21**	0.59	**80.03**	0.36	81.20	0.34
Methods	Kapferer TailorShop		CS Aarhus			
	*θ*_*ave*_	*θ*_*stdev*_	*θ*_*ave*_	*θ*_*stdev*_		
Random immunization	80.74	0.94	82.73	0.65		
AV	80.95	0.75	82.81	0.32		
TIM	81.25	0.70	82.82	0.53		
SpreadingDegree	81.30	0.69	82.88	0.46		
MultiplexShield	**82.41**	0.89	**83.11**	0.51
  Under *Layer-Based Protection Scheme*, the average result of percentage of survived nodes at the end of epidemics shows the higher effectiveness of the MultiplexShield, as depicted in Table 6. We know that the layer-based protection scheme has similar properties of structure with single-layered except the stochastic epidemic transmission probability.

**Table 6:** Layer-based protection scheme, targeted high-degree nodes attack scenario

Methods	Florentine Families		Krackhardt HighTech		Vicker 7thGrader	
	*θ*_*ave*_	*θ*_*stdev*_	*θ*_*ave*_	*θ*_*stdev*_	*θ*_*ave*_	*θ*_*stdev*_
Random immunization	77.03	3.12	74.84	1.55	73.80	1.91
AV	77.09	1.99	73.92	2.08	73.78	1.81
TIM	87.78	0.90	75.08	1.33	74.16	1.49
SpreadingDegree	**87.88**	1.02	75.11	1.41	74.77	1.18
MultiplexShield	85.66	2.31	**76.13**	1.43	**76.06**	1.75
Methods	Lazega LawFirm		Physician Innovation		C.Elegans	
	*θ*_*ave*_	*θ*_*stdev*_	*θ*_*ave*_	*θ*_*stdev*_	*θ*_*ave*_	*θ*_*stdev*_
Random immunization	72.94	0.90	74.36	0.45	74.56	0.41
AV	73.92	0.57	74.78	0.34	74.31	0.34
TIM	74.36	0.66	75.13	0.66	75.88	0.33
SpreadingDegree	74.37	0.94	75.18	0.47	76.11	0.26
MultiplexShield	**75.14**	0.70	**76.03**	0.31	**76.60**	0.42
Methods	Kapferer TailorShop		CS Aarhus			
	*θ*_*ave*_	*θ*_*stdev*_	*θ*_*ave*_	*θ*_*stdev*_		
Random immunization	75.46	1.01	78.72	0.47		
AV	76.03	1.16	78.73	0.49		
TIM	76.47	0.80	78.77	0.53		
SpreadingDegree	76.53	0.91	78.80	0.71		
MultiplexShield	**77.32**	0.92	**79.58**	0.69

### Scalability evaluation

Let us recall our second evaluation goal, which aims to measure how scalable is the proposed MULTIPLEXSHIELD with respect to the changing of graph size and different *k* budget size. Here we report the result of scalability evaluation. All of the experiments were simulated on the same machine with Intel i5-2520M CPU *@* 2.50GHz x 4 and 4 GB memory, running Linux (2.6 kernel) 64-bit. The computational running time is used for this scalability evaluation of MULTIPLEXSHIELD with respect to *n* (number of nodes) and *m* (number of edges), respectively. We evaluate on multiplex graph consisting of synthetic random graph in such a way that we can change the number of nodes but still maintain the number of edges and vice versa. Different values of *k* were used to evaluate the scalability in different scale of protection set.

To perform simulation by changing the number of nodes with a fixed number of edges (and vice versa), we generate multiplex networks using Erdos Renyi *G*(*n*,*m*) model (Erdos and Renyi 1959). The degree distribution of each generated layer follows the normal distribution.

**Fig. 3:**
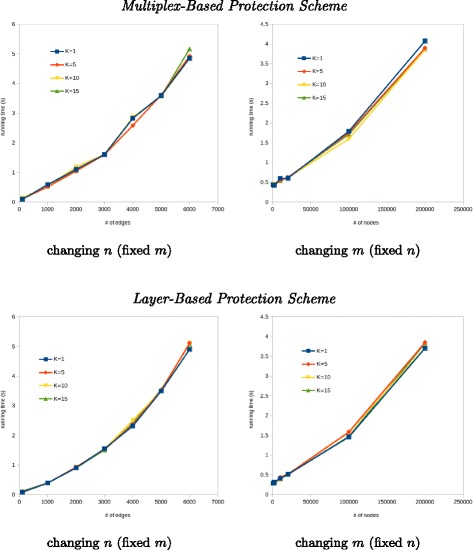
Scalability evaluation of proposed MULTIPLEXSHIELD

From Fig. 3, we can infer the scalability of MULTIPLEXSHIELD in two different protection schemes. Firstly, in both of multiplex-based and layer-based scheme, the running time of MULTIPLEXSHIELD scales linearly with respect to the number of nodes. In left subfigures of Fig. 3, we illustrate the changes of the number of nodes (*n*) and fix the number of edges (*m*=10,000) in a 3 layer multiplex graph. The number of nodes is changed from *n*={100;1000;2000;3000;4000;5000;6000}. The average degree of each layer is {99.00;20.00;10.00;6.67;5.00;4.00;3.33} Secondly, we change *m* and fix *n*=1,000 in a 3 layer graph, as shown in the right subfigures. The number of edges is changed from *m*={1000;2000;10,000;20,000;100,000;200,000}. The average degree of each layer is {2.00;4.00;20.00;40.00;200.00;400.00} It can be inferred that MULTIPLEXSHIELD scales linearly with respect to the number of edges in both multiplex-based and layer-based scheme. Hence, the MULTIPLEXSHIELD scalable with the changing of graph size, which means it is suitable for large graphs.

## Discussions

We have analyzed and evaluated our proposed methods to suppress the propagation of epidemic in multiplex social networks. In substance, our approaches may have limitations which rely on two essential assumptions: static network and pre-emptive protection scheme.

Firstly, we assume that the underlying network structure is static and remains unchanged as the contamination spreading arises. However, real-world social networks evolve dynamically with some users joining and leaving the networks, and relationships among users being formed and removed over time. This condition requires more complex analysis and modeling to incorporate the network evolution.

Secondly, we focus on the pre-emptive scheme by protecting the most critical nodes in a network before the epidemic started, aiming to inhibit its spreading. Given a limited *k* budget, we spend all the available budget prior any infection occurs. Despite its effective prevention, in existing large social networks such as Facebook and Twitter, protecting a particular set of nodes adaptively against the occurring contamination is more realistic. Instead of determining the set of protected nodes in a single time point, we can gradually select a node to respond adaptively the new incoming contaminant which changes over time. In this setting, a different contaminant may receive different protection scheme as a response.

Therefore, extending our algorithms into an adaptive scheme and accommodating the temporal dynamics of propagation into our analysis and model will become an interesting future direction.

## Conclusions

In this paper, we have addressed the problem of suppressing the epidemic propagation in multiplex social networks using the pre-emptive spectral graph protection. We consider the role of graph spectral properties, degree centrality and layer-wise stochastic propagation rate to pre-emptively select *k* most suitable nodes for protection. Thus, we proposed an effective and scalable algorithm, called MULTIPLEXSHIELD. We have evaluated our proposal in two different approaches: multiplex-based and layer-based node protection schemes. Besides, two kinds of common attacks have also evaluated: random and targeted attack. Results on various real-world multiplex datasets show that our proposed MULTIPLEXSHIELD not only effective but also scalable to suppress the epidemic spreading in multiplex networks.
